# Accuracy of the neurosurgeons estimation of extent of resection in glioblastoma

**DOI:** 10.1007/s00701-019-04089-8

**Published:** 2019-10-28

**Authors:** Sümeyye Sezer, Martin J. van Amerongen, Hans H. K. Delye, Mark ter Laan

**Affiliations:** 1 Department of Neurosurgery, Radboud University Medical Center, Geert Grooteplein Zuid 10, 6525 GA Nijmegen, The Netherlands; 2 Department of Radiology, Radboud University Medical Center, Geert Grooteplein Zuid 10, 6525 GA Nijmegen, The Netherlands

**Keywords:** Extent of resection, Glioblastoma multiforme, Interobserver agreement, Tumor volume measurement

## Abstract

**Background:**

The surgeons’ estimate of the extent of resection (EOR) shows little accuracy in previous literature. Considering the developments in surgical techniques of glioblastoma (GBM) treatment, we hypothesize an improvement in this estimation. This study aims to compare the EOR estimated by the neurosurgeon with the EOR determined using volumetric analysis on the post-operative MR scan.

**Methods:**

Pre- and post-operative tumor volumes were calculated through semi-automatic volumetric assessment by three observers. Interobserver agreement was measured using intraclass correlation coefficient (ICC). A univariate general linear model was used to study the factors influencing the accuracy of estimation of resection percentage.

**Results:**

ICC was high for all three measurements: pre-operative tumor volume was 0.980 (0.969–0.987), post-operative tumor volume 0.974 (0.961–0.984), and EOR 0.947 (0.917–0.967). Estimation of EOR by the surgeon showed moderate accuracy and agreement. Multivariable analysis showed a statistically significant effect of operating neurosurgeon (*p* = 0.01), use of fluorescence (*p* < 0.001), and resection percentage (*p* < 0.001) on the accuracy of the EOR estimation.

**Conclusion:**

All measurements through semi-automatic volumetric analysis show a high interobserver agreement, suggesting this to be a reliable assessment of EOR. We found a moderate reliability of the surgeons’ estimate of EOR. Therefore, (early) post-operative MRI scanning for evaluation of EOR remains paramount.

**Electronic supplementary material:**

The online version of this article (10.1007/s00701-019-04089-8) contains supplementary material, which is available to authorized users.

## Introduction

Glioblastoma (GBM) represents 47.1% of malignant primary brain tumors, making it the most common type of malignant primary brain tumor [1]. Even though survival rates have improved, prognosis of GBM remains poor. Surgery aimed at as much resection as safely possible is the main treatment option. Increasing the extent of resection (EOR) of GBM is associated with prolonged survival [2]. Also, adjuvant radiochemotherapy showed higher survival rates in patients with complete resection (EOR ≥ 90%), compared with partial resection (EOR < 90%) [3].

Using volumetric analysis, it is possible to quantify the EOR using MR imaging. Prior studies using semi-automated methods for this purpose found a high interobserver agreement [4, 5]. However, when manual segmentation is used, a low interobserver agreement in the assessment of tumor resection rates on magnetic resonance imaging (MRI) is described. This applies particularly for post-operative tumor volume and residual tumor volume [6].

Before the general use of post-operative scanning, intra-operative estimation by the neurosurgeon was used to determine partial, subtotal, or total tumor resection. The only study that compared this estimation with the presence of residual tumor mass on a MR image, dates back to 1994 [7]. It showed that neurosurgeons often underestimate the presence of residual tumor. Residual tumor mass was three times more often seen on early post-operative MRI than estimated by the neurosurgeon [7].

With the advancement of microsurgical techniques and the introduction of adjuncts such as fluorescence, we hypothesize a high accuracy of the surgeons’ estimation of the EOR. Possibly, the post-op MRI scan can be omitted after resection using fluorescence. This study aims to compare the EOR estimated by the neurosurgeon with the EOR determined using volumetric analysis on post-operative MR images.

## Methods

### Participants

Because the study implied no burden for the patients, ethical approval was waived by the ethical committee. In this retrospective study, patient characteristic, operation details, and tumor characteristics were prospectively collected. Therefore, no informed consent was required. Adult patients (≥ 18 years old) who underwent a resection for GBM were included. Exclusion criteria were as follows: (1.) patients without a pre-operative contrast-enhanced MR image (i.e., less than 48 h before surgery) or without an early post-operative MRI (i.e., within 72 h after surgery) and (2.) patients that had prior treatment for the target tumor.

### Test methods

All patients were treated according to protocol; neuronavigation was used in all patients and 5-ALA was used for intra-operative fluorescence in 22 patients. The EOR by the neurosurgeon was estimated by the operating surgeon post-operatively (EOR_surgeon_), before the post-operative MR scan was made. This estimation was based on intra-operative microscopic visualization and neuronavigation. Post-operative MR imaging was performed within 72 h after surgery.

Pre- and post-operative T1-weighed MR images (slice thickness 1.0 mm) with and without gadolinium contrast were used for semi-automated segmentation of the tumor. Tumor volumes were calculated through semi-automated volumetric analysis using Brainlab software (Brainlab, iPlanNet version 2.3.1.215.1, Munich, Germany) by three observers (a neurosurgeon, a radiologist, and a trained medical student). Previous literature stated the use of this method to be reliable for tumor measurements [4, 5]. All observers are skilled in interpreting MR images for brain tumors. Each observer was blinded for the data measured by the other observers and for the surgeons’ estimate. The neurosurgeon did not measure the tumors of his own patients. For this reason, there was a second neurosurgeon measuring these patients’ MR images.

Our definition of tumor volume was in accordance with previous descriptions [6]: the contrast enhancing mass, including the possibly present necrotic area. All three observers were instructed to include any tissue with a high post-contrast signal in their estimation. The minimal detectable residual would be one voxel, which corresponds with 0.954 mm^3^ using our settings. To eliminate hemorrhage inclusion in post-operative tumor volume (Post-opTV) determination, any parts around the resection area with high signal on T1-weighed image without contrast were subtracted from the hyperintense mass on the contrast-enhanced T1-weighed image. Extent of resection (EOR) was calculated as:

$$ \mathrm{EOR}\ \left(\mathrm{in}\%\right)=\left(\ \left(\mathrm{Pre}-\mathrm{opTV}-\mathrm{Post}-\mathrm{opTV}\right)/\mathrm{Pre}-\mathrm{opTV}\ \right)\times 100\% $$

This EOR calculation was done for the measurements of the surgeon, the radiologist, and the student, respectively, resulting in an EOR_obs1_, EOR_obs2_, and EOR_obs3_. The mean of these three measurements is defined as the EOR_MRI_.

### Power calculation

Previous literature comparing the presence of residual tumor on MR images with the estimation made by the neurosurgeon is scarce. We predicted to find an accuracy of 80%, meaning that the estimation about the presence of residual tumor mass should agree with the findings on MRI in 80% of the cases. Sample size calculation with a 95% confidence probability, resulted in a sample size of 62.

### Analysis

All analyses were performed on SPSS software version 25 (IBM Inc., Armonk, New York) for Windows (Microsoft Inc., Redmond, Washington). We have used an altered Bland-Altmann plot to assess agreement between more than two observers [8].

Interobserver agreement for pre-opTV, post-opTV, and EOR was measured using intraclass correlation coefficient (ICC) based on a two-way random model for absolute agreement. Using semi-automated volumetric analysis methods, even the smallest remnants are detected. Therefore, a cut-off value of 99% was used as a surrogate for residual tumor only for accuracy calculation.

A univariate multivariable general linear model was used to study the factors influencing the accuracy of EOR_surgeon_. Here, we used Δ = EOR_surgeon_ − EOR_MRI_ as the dependent variable. The following variables were included in analysis: Pre-opTV (mean Pre-opTV of the three observers), blood loss during surgery, use of fluorescence, operating neurosurgeon, and EOR_MRI_. Significance for all analysis was set at a *p* value < 0.05.

## Results

### Participants

**Fig. 1:**
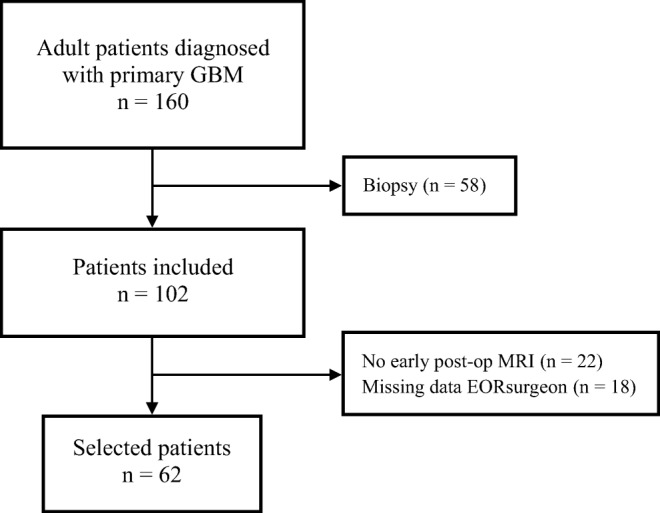
Flowchart describing the selection process of the participants between November 2012 and May 2018. GBM, glioblastoma. Neurosurgeon, extent of resection estimated by the operating surgeon

Between November 2012 and May 2018, 62 patients were included in the study. A flowchart of the selection process can be found in Fig. 1. The characteristics of the study group are summarized in Table 1. The group consisted of 38 male (61%) and 24 female (39%) patients. The median age at diagnosis was 63.5 year (range 27–78 years).

**Table 1:** Patient characteristics (*n* = 62)

Variable	No. of patients (%)	
Sex		
Male	38 (61)	
Female	24 (39)	
Age at diagnosis		
Median		63.5
Range		27–78
Side of tumor		
Left	32 (52)	
Right	30 (48)	
Tumor location		
Frontal lobe	18 (29)	
Temporal lobe	28 (45)	
Parietal lobe	13 (21)	
Occipital	2 (3)	
Basal ganglia	1 (2)	
Necrosis		
No	10 (16)	
Yes	52 (84)	
Fluorescence guided resection (use of 5-ALA)		
No	40 (65)	
Yes	22 (35)	
Use of neuronavigation		
No	0 (0)	
Yes	62 (100)	
Blood loss (mL)		
Median		200
Range		20–1300
Pre-operative tumor volume* (cm^3^)		
Median		33.7
Range		1.0–169.8

Numbers are absolute values (percentages)

*The mean pre-operative volume of neurosurgeon, radiologist, and medical student

### Agreement between EOR_obs1_, EOR_obs2_, and EOR_obs3_

The altered Bland-Altmann plot for pre-opTV and post-opTV showed good agreement between observers (see Supplemental Digital Content). An ICC of 0.980 (95% CI 0.969–0.987) for pre-opTV and 0.974 (95% CI 0.961–0.984) for post-opTV showed an excellent reliability between observers for these measurements [9].

**Fig. 2:**
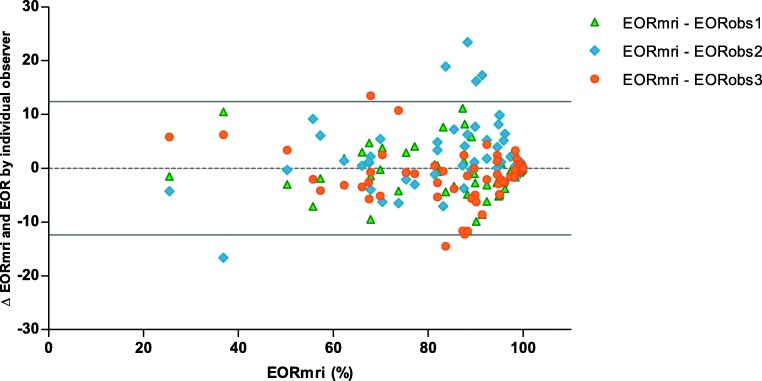
Bland-Altman plot for three observers [8]. EORmri (*x*-axis) is plotted against EORmri minus the EOR measured by the individual observer (*y*-axis). This graph displays per patient the agreement between the three observers. High agreement corresponds with the three colors being close together. The lines represent the limits of agreement. EOR, extent of resection; EORmri, mean EOR of three observers; EORobs1, EOR measured by neurosurgeon; EORobs2, EOR measured by radiologist; EORobs3, EOR measured by student

The Bland-Altmann plot for EOR can be found in Fig. 2. The estimated limits of agreement were – 12.39 to 12.39. The plot shows no systematic over or underestimation of EOR by any observer. There was an excellent reliability for EOR measurement, with an ICC of 0.947 (0.917–0.967) (Table 2).

**Table 2:** Measurements of observers and corresponding intraclass correlation coefficients

	Neurosurgeon	Radiologist	Student	ICC (95% CI)
Mean pre-opTV (SD)	38.15 (33.47)	37.72 (29.43)	39.92 (33.08)	0.980 (0.969–0.987)
Mean post-opTV (SD)	6.01 (12.01)	5.69 (8.25)	5.68 (10.71)	0.974 (0.961–0.984)
Mean EOR (SD)	86.41 (17.66)	83.21 (16.06)	86.96 (17.96)	0.947 (0.917–0.967)

The ICC was calculated using a two-way random model for absolute agreement. Pre-opTV, post-opTV, and EOR all showed a high ICC, indicating an excellent reliability between observers. *ICC*, intraclass correlation coefficient; *Pre-opTV*, pre-operative tumor volume; *Post-opTV*, post-operative tumor volume; *EOR*, extent of resection

### EOR_surgeon_, EOR_MRI_

**Fig. 3:**
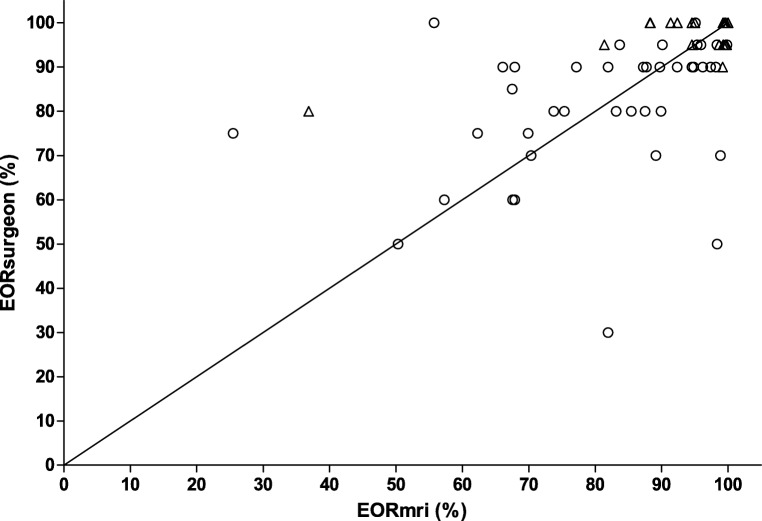
Scatterplot for extent of resection (EOR) based on semi-automatic calculation on MR (EORmri) versus EOR estimated by the operating surgeon (EORsurgeon). Triangles represent resections where fluorescence was used, circles represent resections without fluorescence. The identity line (*y* = *x*) is shown for reference

There is a higher agreement between EOR_surgeon_ and EOR_MRI_ with increasing resection percentage and when fluorescence is used (Fig. 3). The cases are equally distributed above and below the identity line, so there was no systematic EOR over or underestimation by the surgeon.

Presence of residual tumor (defined as EOR < 99%) was estimated by the neurosurgeon almost as often as it was seen on the MRI: surgeons expected residual tumor presence in 76% of the patients, whilst residual was seen in 79% of the MR images (Table 3). The accuracy of predicting presence of residual tumor (defined as EOR < 99%) was 77%, meaning that 77% of the estimations about presence of residual tumor was correct.

**Table 3:** Assessment of completeness of tumor resection: comparison between the neurosurgeon’s estimation and the findings on MRI

Residual tumor	Neurosurgeon (%)	MRI (%)
Yes (EOR < 99%)	76	79
No (EOR ≥ 99%)	24	21

The ICC for EOR_surgeon_ and EOR_MRI_ was 0.641 (95% CI 0.404–0.784). The latter indicates a moderate reliability of the EOR_surgeon_ [9].

Multivariable analysis showed a statistically significant effect of operating neurosurgeon (*p* = 0.01), use of fluorescence (*p* < 0.001), and EOR_MRI_ (p < 0.001) on Δ_surgeon - MRI_. Blood loss and pre-operative tumor volume were not statically significant factors.

Following these findings, an estimated marginal means was calculated for use of fluorescence. Use of fluorescence showed an estimated marginal mean of 12.48 (95% CI 7.26; 17.71), meaning with the use of fluorescence, there is a significant overestimation by the surgeon.

## Discussion

Our main purpose in this study was to compare the EOR estimated by the neurosurgeon post-operatively (EOR_surgeon_) with the EOR determined using volumetric analysis on the post-operative MR scan (EOR_MRI_).

In order to achieve this, we first studied the reliability of semi-automated volumetric analysis for determining EOR. There was a very high interobserver agreement between the three observers for measurements of pre-operative tumor volume, post-operative tumor volume, and extent of resection. Therefore, semi-automatic volumetric measurement of extent resection of glioblastoma is reliable.

Our data shows that residual tumor (defined as EOR < 99%) is seen on MR images as often as suggested by the neurosurgeon. This simple comparison shows an evident result and this way of reporting data is similar to that of the previous study on this subject [7]. However, with the increased precision of statistical analysis in this era, we added a more statistical approach to display our findings. So, we found an accuracy of 77% and a moderate reliability [9].

Higher resection percentage contributed to a higher agreement between EOR_surgeon_ and EOR_MRI_. The use of fluorescence also contributed to a higher agreement, with a tendency of overestimation of the EOR by the surgeon (marginal means 12.48 95% CI 7.26; 17.71). The reliability of the surgeons’ estimate was surgeon dependant.

Using manual segmentation, others have reported high interobserver agreement only for pre-operative tumor volume, but not regarding post-operative volume and EOR [6]. Prior studies using semi-automated methods found a high interobserver agreement for calculation of resection percentage [4, 5]. Our results agree, showing that semi-automatic volume assessment in GBM is a reliable method for detecting post-operative residual and determining EOR.

Only one prior study has reported on the reliability of surgeons’ estimate of EOR [7]. In this study, data collection was nominal: presence of residual tumor was answered with “yes,” “no,” or “?.” Residual tumor was detected 3 times more often on MRI than estimated by the surgeon. Our data shows the findings of residual tumor on MRI to be equal to the estimation of the neurosurgeon. At first hand, this seems as an improvement in reliability of surgeons’ estimate as hypothesized, but further analysis shows only moderate reliability.

The strength of our study is the use of semi-automatic volumetric assessment of EOR and showing this method of determining EOR is highly reliable. Another strength is the statistical assessment of reliability of EOR_surgeon_. We found an equal distribution of over and underestimation of EOR by the surgeon, so we did not confirm the systematic overestimation of the surgeon as suggested by others [7]. We did find that accuracy of the surgeons’ estimate is surgeon dependant. So, some surgeons estimate the resection percentage better than others. Even though the use of fluorescence resulted in a slight overestimation, it did increase the accuracy of surgeons’ estimate.

There were a few limitations in our study. Our study was powered to detect accuracy of 80%, but the low number of subjects limits the factors that can be included in our multivariate analysis. We have calculated accuracy in an indirect way, using a dichotomy with 99% EOR as the cut-off value. Nevertheless, the different statistical approaches in this study all show a comparable result: moderate agreement for surgeons’ estimate and volumetric assessment by MRI. Finally, since residual volume is clinically more relevant than percentages, we have plotted surgeons estimating complete resection against residual volume. There was no estimation of complete resection when residual was above 6 mL. With minimal residual volume (< 2 mL, 2–4 mL, and 4–6 mL), 17 to 36% of surgeons estimated a complete resection (Supplemental Digital Content, Fig. 3).

## Conclusion

Semi-automatic volumetric analysis showed a high interobserver agreement and should therefore be considered the gold standard for assessment of EOR. The introduction of fluorescence has resulted in better resections [10]. We found it to increase the accuracy of the surgeons’ estimate of fluorescence, whilst resulting in a tendency towards overestimation. Even though surgeons’ estimate of extent of resection has clearly improved since the report of Albert et al., the reliability of their estimation is statistically moderate. Therefore, early post-operative MRI scanning for evaluation of EOR remains paramount.

## Electronic supplementary material

ESM 1 (PDF 147 kb)
