# Supplementary material for: Accuracy of the neurosurgeons estimation of extent of resection in glioblastoma
Source: Acta Neurochir (Wien). 2019 Oct 28;162(2):373–8. doi: 10.1007/s00701-019-04089-8 (PMC6982640; doi:10.1007/s00701-019-04089-8)
Supplement: Supplementary file 1 — (PDF 147 kb) [file 701_2019_4089_MOESM1_ESM.pdf]

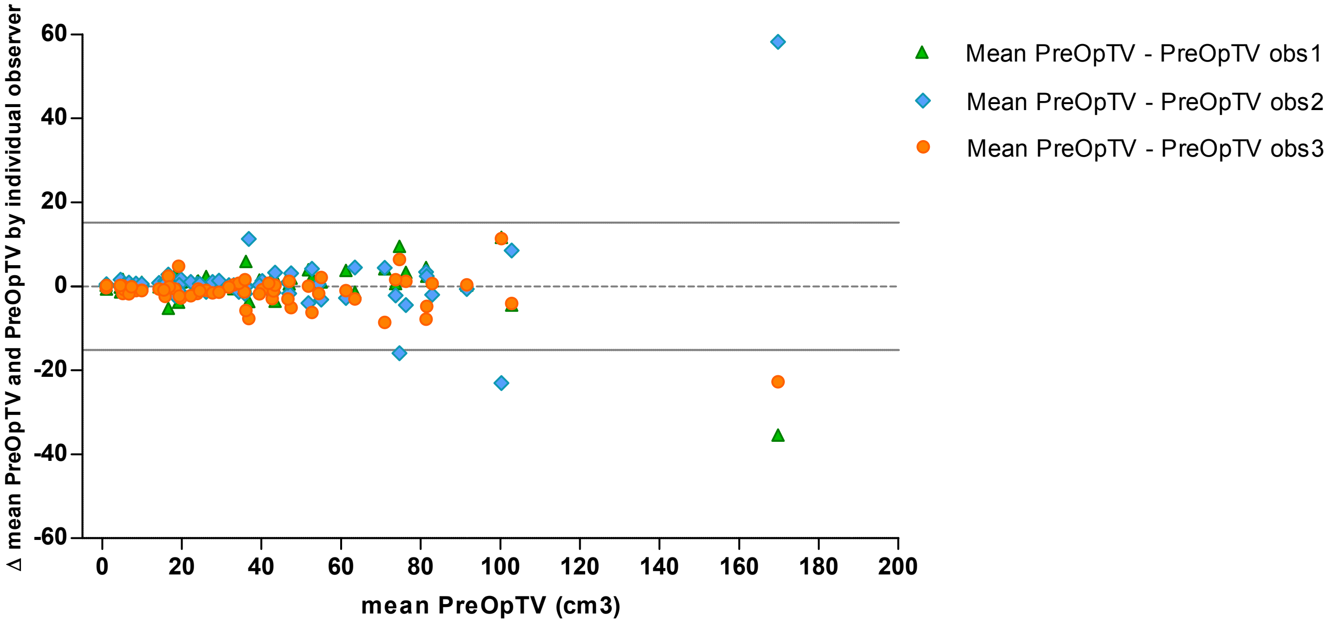

Δ mean PreOpTV and PreOpTV by individual observer
60
40
20
0
-20
-40
-60
0
20
40
60
80
100
120
140
160
180
200
mean PreOpTV (cm3)
Mean PreOpTV - PreOpTV obs1
Mean PreOpTV - PreOpTV obs2
Mean PreOpTV - PreOpTV obs3

Figure 1.

Bland-Altman plot for three observers. Mean PreOpTV (x-axis) is plotted against mean PreOpTV minus the PreOpTV measured by the individual observer (y-axis). This graph displays per patient the agreement between the three observers. High agreement corresponds with the three colours being close together. The lines represent the limits of agreement. PreOpTV: pre-operative tumor volume. Obs1, measured by neurosurgeon. Obs2, measured by radiologist. Obs3, measured by student.

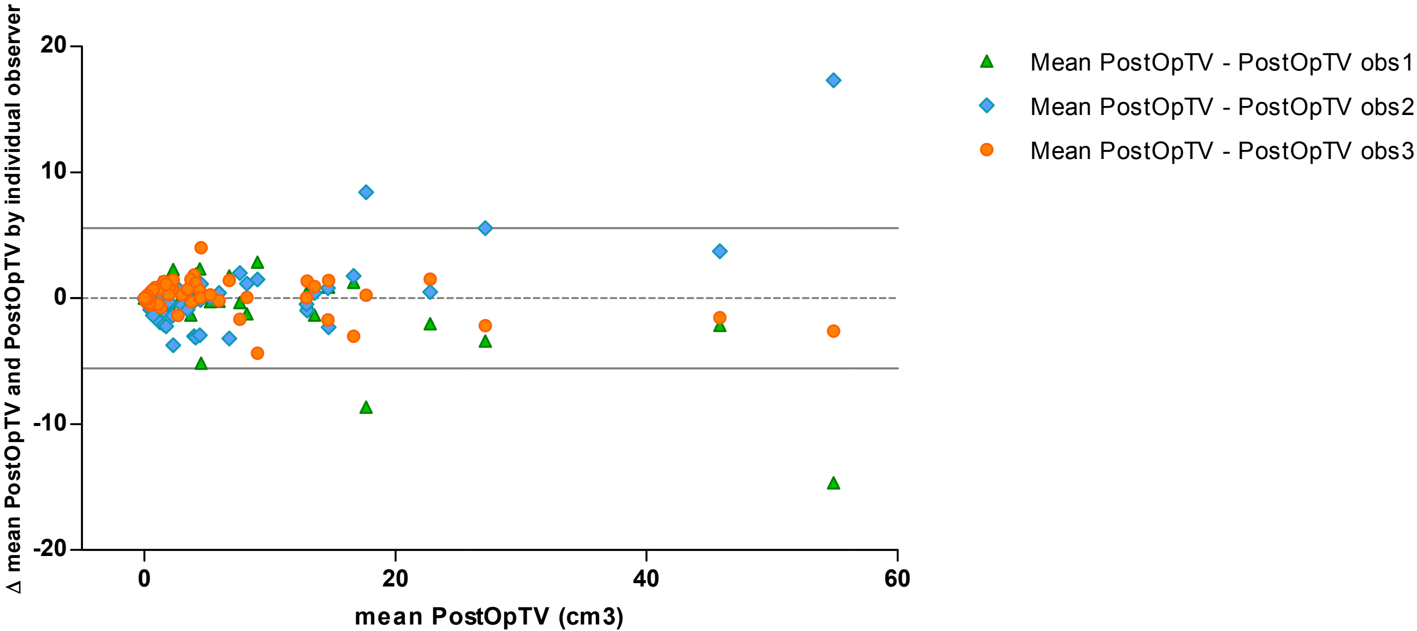
Δ mean PostOpTV and PostOpTV by individual observer
20
10
0
-10
-20
0
20
40
60
mean PostOpTV (cm3)
Mean PostOpTV - PostOpTV obs1
Mean PostOpTV - PostOpTV obs2
Mean PostOpTV - PostOpTV obs3

Figure 2.

Bland-Altman plot for three observers. Mean PostOpTV (x-axis) is plotted against mean PostOpTV minus the PostOpTV measured by the individual observer (y-axis). This graph displays per patient the agreement between the three observers. High agreement corresponds with the three colours being close together. The lines represent the limits of agreement. PostOpTV: post-operative tumor volume. Obs1, measured by neurosurgeon. Obs2, measured by radiologist. Obs3, measured by student.

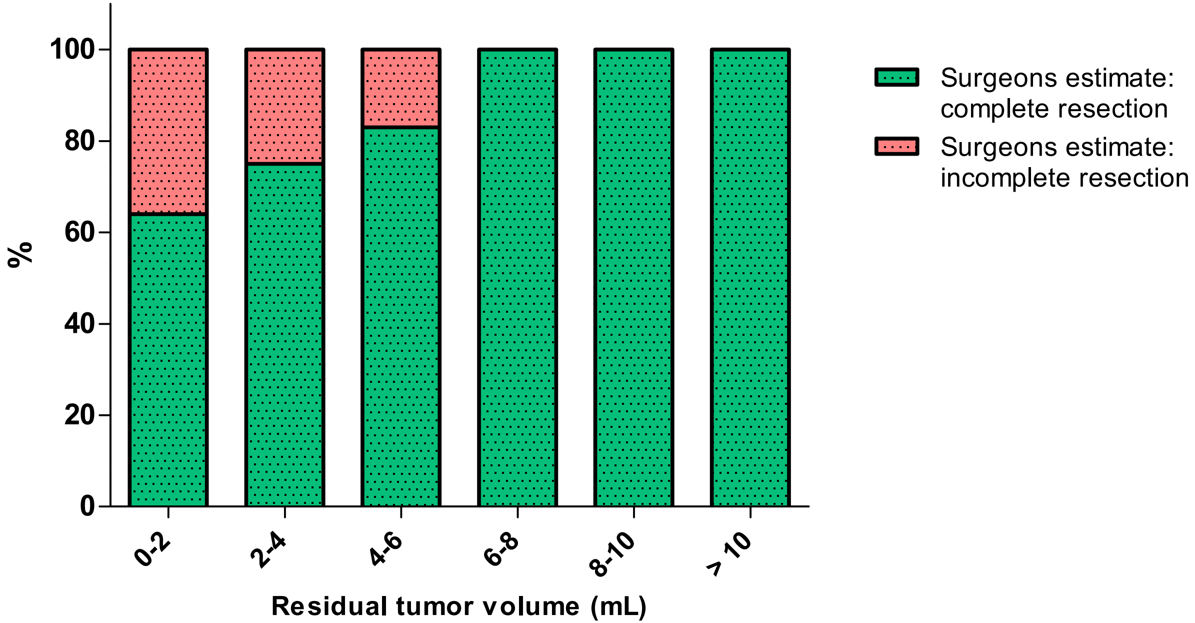

100
80
60
40
20
0
%
0-2
2-4
4-6
6-8
8-10
> 10
Residual tumor volume (mL)
Surgeons estimate: complete resection
Surgeons estimate: incomplete resection

Figure 3.

Amount of residual tumor (x-axis) plotted against the distribution of surgeons estimation (y-axis). This graph shows estimation of the neurosurgeon to be 100% correct when residual volume is higher than 6 cm3.
